# Arctigenin prevents the progression of osteoarthritis by targeting PI3K/Akt/NF‐κB axis: In vitro and in vivo studies

**DOI:** 10.1111/jcmm.15079

**Published:** 2020-02-24

**Authors:** Shangkun Tang, Weijun Zhou, Xinyang Zhong, Jianchen Xu, Huasong Huang, Xinnan Zheng, Jingkang Zhang, Shuyue Yang, Ping Shang, Qian Tang, Haixiao Liu

**Affiliations:** ^1^ Department of Orthopaedic Surgery The Second Affiliated Hospital and Yuying Children's Hospital of Wenzhou Medical University Wenzhou China; ^2^ Department of Clinical Medicine Second Clinical Medical College Wenzhou Medical University Wenzhou China; ^3^ Department of Rehabilitation The Second Affiliated Hospital and Yuying Children's Hospital of Wenzhou Medical University Wenzhou China; ^4^ Department of Orthopedic Surgery Shanghai Jiao Tong University Affiliated Sixth People's Hospital Shanghai China

**Keywords:** arctigenin, chondrocyte, inflammation, NF‐κB, osteoarthritis

## Abstract

Osteoarthritis (OA), which is principally featured by progressive joint metabolic imbalance and subsequent degeneration of articular cartilage, is a common chronic joint disease. Arctigenin (ATG), a dietary phyto‐oestrogen, has been described to have potent anti‐inflammatory effects. Nevertheless, its protective effects on OA have not been clearly established. The target of our following study is to evaluate the protective effects of ATG on IL‐1β–induced human OA chondrocytes and mouse OA model. Our results revealed that the ATG pre‐treatment effectively decreases the level of pro‐inflammatory mediators, such as prostaglandin E2 (PGE2), nitrous oxide (NO), inducible nitric oxide synthase (iNOS), cyclooxygenase‐2 (COX‐2), IL‐6 and tumour necrosis factor alpha (TNF‐α) in IL‐1β–induced human chondrocytes. In addition, ATG protects against the degradation of extracellular matrix (ECM) under the stimulation of IL‐1β and the possible mechanism might be connected with the inactivation of phosphatidylinositol‐3‐kinase (PI3K)/Akt/nuclear factor‐kappa B (NF‐κB) axis. Furthermore, a powerful binding capacity between ATG and PI3K was also uncovered in our molecular docking research. Meanwhile, ATG may act as a protector on the mouse OA model. Collectively, all these findings suggest that ATG could be utilized as a promising therapeutic agent for the treatment of OA.

## 1 INTRODUCTION

Osteoarthritis (OA) is the leading cause of disability worldwide, and in the United States, the number of patients is more than 22.7 million. Most patients have been previously described to experience arthritis‐attributable activity restrictions without any valid treatments except total joint replacement in late stage.^1^, ^2^, ^3^ Moreover, the incidence and scale of OA is predicted to have been continuously increasing in the following years owing to the ripening of the population, ascending obesity rates and a large amount of traumatic joint injuries.^4^, ^5^, ^6^ Its pathological changes are commonly characterized by the progressive cartilage loss, synovial inflammation, subchondral bone remodelling and osteophyte formation.^7^ As a further comment, accumulating evidence has shown that inflammatory mediators, particularly interleukin‐1 beta (IL‐1β), play a significant role in OA progress via promoting the secretion of many pro‐inflammatory factors or catabolic enzymes.^8^ Meanwhile, some researchers have found that the level of IL‐1β is dramatically elevated in the synovial liquid, the articular cavity and the serum of OA individuals.^9^, ^10^, ^11^ Likewise, the secretion of cartilage catabolic enzymes could be up‐modulated by the stimulation of IL‐1β in vitro. Among them, metalloproteinases (MMPs) and a disintegrin and metalloproteinase with thrombospondin motifs (ADAMTS) function as the main destroyers of extracellular matrix (ECM).^12^ Therefore, strategies aiming at the inhibition of IL‐1β itself or IL‐1β–induced inflammatory responses hold promise in slowing down the progression of OA.

Prior studies have noted the importance of NF‐κB on pathogenesis and progression of OA.^13^, ^14^ When under normal situation, NF‐κB is situated in the cytoplasm as an inactive form, connected with IκBα, an inhibitory subunit.^14^, ^15^ When stimulated by IL‐1β, IκBα is phosphorylated by IκB kinases (IKKs) and subsequently degraded in the cytoplasm. Afterwards, NF‐κB p65 molecule is separated with IκBα and experiences a translocation from the cytoplasm to the nucleus, which further modulated the translation of related downstream devastative gene, such as iNOS, COX‐2, MMPs and ADAMTs.^12^, ^15^, ^16^, ^17^ PGE2, activated by COX‐2, as an inflammation‐related factors during the progress of OA can highly promote the expression of ADAMTS5 and MMPs, which attribute to the of ECM in cartilage tissue.^18^ Moreover, NO, as an inflammatory mediator, belongs to the nitric oxide synthase (NOS) family of enzymes, with which MMP production and many other pro‐inflammatory factors are up‐regulated.^19^ Type II collagen and aggrecan are the major structural constituents of the ECM,^12^, ^20^ while MMP13, as a vital part of collagenase family, occupies an extremely significant position aiming type II collagen disintegration, and ADAMTS5, as a key member of secreted zinc proteases, shows dramatically destructive effects of aggrecan synthesis.^21^, ^22^ Moreover, a specific molecular repressor in the NF‐κB p65 nuclear translocation has been verified to delay the process of OA in the mouse OA models.^23^ On the other hand, the PI3K/Akt signalling pathway has been expounded to be involved in the adjustment of NF‐κB abnormal activation.^24^, ^25^, ^26^, ^27^ Briefly, the phosphorylation of the p85 regulatory subunit of PI3K serves activation of PI3K signalling and triggering AKT phosphorylation, which then binds multiple downstream target proteins such as mTOR, NF‐kB, GSK‐3beta. Among them, to conclude, NF‐kB is the most important hinge in inflammatory diseases. The inhibition of PI3K/Akt/NF‐κB axis is expected to be a strategy with high efficiency for attenuating the development of OA.

Although there are already drugs for OA in the market, such as sodium hyaluronate, NASIDs, glucosamine, chondroitin sulphate, most of them were proved to relive the pain of joint, but whether it could truly prevent the progression of OA is still debatable and many side effects were also reported.^28^, ^29^, ^30^, ^31^, ^32^, ^33^ Thus, our present study is trying to find a safer agent‐derived natural plant, which could truly attenuate OA with little side effects. Arctigenin (ATG), a phenylpropanoid dibenzylbutyrolactone lignan, is pulled from the seed of *Arctium lappa L* (*A lappa*), which is commonly known as greater burdock, a kind of edibles worldwide.^34^ More and more researchers have subscribed to the view that ATG possesses immeasurable pharmacological value, including antioxidant, neuronal protection, antiviral and anti‐inflammatory effects.^35^, ^36^, ^37^, ^38^ The anti‐inflammatory effect has been confirmed on LPS‐induced inflammation models in RAW264.7 cells or human U937 macrophage cells by means of restraining NF‐κB, JAK‐STAT and MAPK pathway.^39^, ^40^, ^41^, ^42^ Additionally, a recent study has exposited that ATG remits LPS‐induced acute lung injury in rats.^43^ To the most important, the ATG was found to exhibit the ability to protect against rheumatoid arthritis by target Akt/NF‐κB.^44^ All of these may lead to the conclusion that ATG might own a potential therapeutic effect on the developmental process of OA and there is an imperative to figure out the underlying mechanisms.

## 2 MATERIALS AND METHODS

### 2.1 Reagents and antibodies

Arctigenin (purity > 98%) was purchased from the Tianjin Shilan Technology Company. Cell culture reagents were purchased from Gibco (Life Technologies Corp.). Cell Counting Kit‐8 (CCK‐8) was bought from DOJINDO (Kumano, Japan). Recombinant human IL‐1β protein was supplied by Novoprotein. Type II collagenase, sodium carboxymethyl cellulose (CMC‐Na) and dimethyl sulphoxide (DMSO) were received from Solarbio. The primary antibody against Lamin B and GAPDH was obtained from Abcam, iNOS antibodies were obtained from Sigma‐Aldrich, and goat anti‐rabbit and antimouse IgG‐HRP were from Bioworld (OH). The anti‐COX‐2, anti‐p65, anti‐IκBα, anti‐PI3K, anti‐p‐PI3K, anti‐Akt and anti‐p‐Akt antibodies were acquired from Abcam. The second antibody (Alexa Fluor® 488‐labelled goat anti‐rabbit IgG) was achieved from Yeasen. The 4', 6‐diamidino‐2‐phenylindole (DAPI) was purchased from Beyotime.

### 2.2 Primary human chondrocyte culture

Following the standards of the terms of the Medical Ethical Committee of the Second Affiliated Hospital, Wenzhou Medical University (ethic cord: LCKY‐2017‐37), and adhering to the guidelines of the Declaration of Helsinki,^45^ the human articular cartilage samples with OA were collected from 10 eligible patients, and only patients undergoing the total knee arthroplasty aged between 62 and 68 years were included in the study (five men and five women). All patients signed the informed consent. The diagnosis of OA matches the classification criteria of the American College of Rheumatology (ACR).^46^

The hyaline cartilages of collected tissue were rinsed in PBS and cut up into pieces and subsequently digested with collagenase II (2 mg/mL) in DMEM/F12 at the temperature of 37°C for 4 hours. Afterwards, chondrocytes were seeded in a 25‐cm^2^ task at a density of 2 × 105 cells/mL in complete DMEM/F12 medium (with 10% FBS and 1% antibiotic) in a 5% CO_2_ atmosphere at 37°C. To avoid the phenotype loss of chondrocytes, cells at least in two passages were employed for subsequent experiments.

### 2.3 Cell viability

The toxicity of ATG towards human OA chondrocytes was determined via the CCK‐8 kits obeying the protocols of the manufacturer. Firstly, the chondrocytes in the second passage were cultured in 96‐well plates (50 000 cells/cm^2^) with serum‐free culture medium for 24 hours. Then, the cells were incubated with respective concentrations of ATG (5, 10 and 50 μmol/L) for the duration of 24 and 48 hours. At the appointed time, followed by rinsing in PBS thrice, the cells were disposed with 100 µL 10% of CCK‐8 solution (diluted in serum‐free DMEM/F12) and subsequently incubated at the temperature of 37°C for 2 hours. The medium was then collected and assessed at 450 nm by a micro‐plate reader. The experiment was performed five times.

### 2.4 Nitrous oxide measurement and ELISA

The activity of NO in culture medium was appraised via the Griess reagent as previous descriptions did.^47^ According to the manufacturer's introductions, the PGE2, TNF‐α, IL‐6, collagen II, aggrecan, MMP13 and ADAMTS‐5 level in culture medium were assessed using the ELISA kit (R&D Systems). The experiment was performed five times.

### 2.5 Western blotting

The total protein was gained from cells using the RIPA lysis buffer with 1% phenylmethanesulphonyl fluoride (PMSF) on the ice bath for 10 minutes, and subsequent centrifugation (15 338 *g*) was at 4°C for 20 minutes. Later, the concentrations of protein were detected by virtue of the BCA protein assay kit. The protein (30 mg) of the respective group was separated by sodium dodecyl sulphate (SDS)‐polyacrylamide gel electrophoresis, followed by transferring to a polyvinylidene difluoride (PVDF) membrane (Millipore). After being blocked with 5% BSA for 2 hours, the obtained membranes were then incubated with the primary antibodies: Lamin B (1:5000), AKT (1:1000), p65 (1:1000), PI3K (1:1000), IκBα (1:1000), COX‐2 (1:1000), GAPDH (1:5000), P‐PI3K (1:1000), P‐AKT (1:1000) and iNOS (1:1000) overnight at 4°C. At the appointed time, the membranes were treated with HRP‐labelled secondary antibodies for 2 hours at indoor temperature. After rinsing in TBST for three times, the blots were visualizable by virtue of the electrochemiluminescence plus reagent (Invitrogen), and the intensity of blots was quantified with Image Lab 3.0 software (Bio‐Rad).

### 2.6 Real‐time PCR

The total RNA from the human chondrocytes was gained through TRIzol reagent (Invitrogen), and the procedures obey the manufacturer's guidelines. The first‐strand complementary DNA (cDNA) was synthesized by dint of 1000 ng of total RNA and the QuantiTect Reverse Transcription Kit. As for the quantitative real‐time PCR (qPCR), the reactions were acted in a 10 μL system (diluted cDNA (4.5 μL), reverse primer (0.25 μL), forward primer (0.25 μL) and SYBR (5 μL) Green Master Mix) through the CFX96 Real‐Time PCR System (Bio‐Rad Laboratories) and the parameters were set up as follows: 10 minutes at 95°C, 40 cycles of 15 seconds at 95°C and 1 minutes at 60°C. The collected cycle threshold (Ct) values were normalized to the expression of GAPDH. The level of relating mRNA of related genes was measured by the 2^−△△^Ct method.^48^ The NCBI Primer‐Blast tool (https://www.ncbi.nlm.nih.gov/tools/primer-blast/) was applied to design and form the necessary primers, and the sequences are shown in Table 1.

**Table 1:** The primer sequences used for RT‐PCR

Gene	Forward primer	Reverse primer
iNOS	5′‐CCTTACGAGGCGAAGAAGGACAG‐3′	5′‐CAGTTTGAGAGAGGAGGCTCCG‐3′
COX‐2	5′‐GAGAGATGTATCCTCCCACAGTCA‐3′	5′‐GACCAGGCACCAGACCAAAG‐3′
TNF‐α	5′‐GTCAGATCATCTTCTCGA ACC‐3′	5′‐CAGATAGATGGGCTCATACC‐3′
IL‐6	5′‐GACAGCCACTCACCTCTTCA‐3′	5′‐TTCACCAGGCAAGTCTCCTC‐3′

### 2.7 Immunofluorescence

The cells were planted in a 25‐mm glass in 6‐well plate and incubated with serum‐starved medium overnight. Subsequently, the cells were solely treated with IL‐1β (10 ng/mL) or co‐treated with ATG (50 μmol/L) and IL‐1β (10 ng/mL) for 24 hours. However, the incubation time of the immunofluorescence staining of p65 with IL‐1β was declined to 2 hours. The glass coverslips cultured with chondrocyte monolayers were firstly rinsed thrice in PBS. Next, cells were treated as follows: fixation in 4% paraformaldehyde at 4℃ for 15 minutes; permeabilization in PBS including 0.5% Triton X‐100 for another 15 minutes at indoor temperature; treatment with 5% goat serum for 1 hour at 37°C; and incubation with primary antibodies against p65 (1:200), collagen Ⅱ (1:200) and MMP‐13 (1:200) for the whole night at the temperature of 4°C. At the adjacent day, cells were exposed to Alexa Fluor® 488‐labelled conjugated secondary antibodies (1:400) for 1 hour and DAPI for 1 minute successively. At last, cell samples were observed under fluorescence microscopy (Olympus Inc). The ImageJ software 2.1 (Bethesda) was applied to assess the fluorescence intensity by observers who were blinded to the experimental groups.

### 2.8 Molecular modelling

The structure of ATG and PI3K, downloaded from Chemicalbook (https://www.chemicalbook.com/) and RCSB PDB (https://www.rcsb.org/; PDB ID: http://www.rcsb.org/pdb/search/structidSearch.do?structureId=3LJ3), respectively, was applied for molecular docking study and reconstructed in PyMoL software (version 1.7.6). For the protein‐ligand docking analysis, the AutoDockTools (version 1.5.6) was applied. The whole of the 3D interactions was generated in PyMoL software, and the 2D image was manufactured by Ligplus (version 1.4).

### 2.9 Mice OA models

Forty‐five eight‐week‐old C57BL/6 male wild‐type mice were acquired from SLAC Laboratory Animal Co., Ltd. The protocol for animal care and use was not only complied with the Guide for the Care and Use of Laboratory Animals of the National Institutes of Health, but also ratified by the Animal Care and Use Committee of Wenzhou Medical University (ethic code: wydw3014‐0127). The destabilization of the medial meniscus (DMM) as previously described 49 was used to establish the mouse OA model. Briefly, the mice were anaesthetized with 2% (w/v) pentobarbital (40 mg/kg, ip); under the direct visualization, the joint capsule of the right knee was exposed followed by transacting the medial to the patella tendon and the medial meniscotibial ligament with an ophthalmic micro‐surgical knife. The sham mice were only received an arthrotomy without ligament cut down. After the operation, the mice were randomly divided into three groups (n = 15): sham control group, an OA group (DMM) and an OA treated with the ATG group (DMM + ATG). Mice in the DMM group received a gavage of vehicle (0.5% CMC‐Na, a dietary cosolvent), while mice in the DMM + ATG group received oral administration of ATG (30 mg/kg, dissolved in CMC‐Na) daily for 8 weeks. This dose of ATG was prepared by adapting the procedure used in the previous study^39^, ^43^, ^50^, ^51^, ^52^, ^53^ and our preliminary experiments. The mice were maintained in a temperature‐controlled room (20 ± 2°C) on a 12:12‐h light‐dark cycle and given free access to water and food. The animals were killed by cervical dislocation at 8 weeks after surgery. The whole knee joints were collected for further evaluation.

### 2.10 Histological assessment

The harvested joint samples were treated as follows: fixation in 4% paraformaldehyde for 1 day and subsequent decalcification in 10% EDTA solution at 4°C for 2 months. Afterwards, the samples were dried using graded ethanol, vitrification by dimethylbenzene and implanting into paraffin slabs. To assess the cartilage tissue degradation, sagittal sequential segments (7 μm thick) of full joints were attained with per joint, which owns 10 slides (70 μm thick) to select. Afterwards, the selected sections went through staining via Safranin O/Fast Green. The stained section images were captured with a microscope. The Osteoarthritis Research Society International (OARSI) (0‐12) of both the medial tibial plateau and femoral condyle was summed to estimate the damage extent to the articular cartilage.^54^ The Likert scale consists of the following scoring grades: 0 signifies typical cartilage, 0.5 = undamaged surface and losing PG, 1 = shallow fibrillation with no cartilage loss, 2 = perpendicular fissures and surface lamina loss, 3 = perpendicular fissures/corrosion for 1/4 of the quadrant thickness of the calcified layer, 4 = lesion spreads to 1/2 of the quadrant thickness of the calcified cartilage, 5 = lesion spreads to 3/4 of the quadrant thickness of the calcified cartilage and 6 = lesion spreads to >3/4 of the calcified cartilage quadrant thickness. Using separate grades allows identification of the severe lesions and the universal extent of damage.

### 2.11 Statistical analysis

All tests were achieved independently fivefold. Outcomes were expressed as means ± SD The variances of two or more groups were analysed using (ANOVA) statistical test. Thereafter, Tukey's test analysed the disparities of the treatment and control groups using SPSS version 20.0. Statistical significance was present for values of *P* < .05.

## 3 RESULTS

### 3.1 Arctigenin did not show toxicity on chondrocyte at the dose of 0‐50 μmol/L

**Figure 1:**
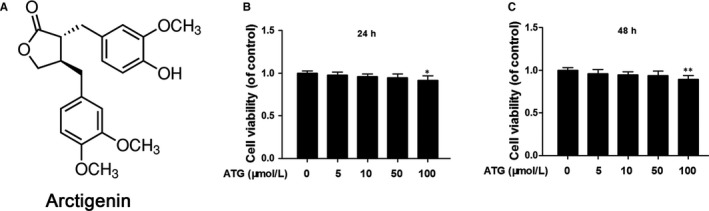
Effects of arctigenin (ATG) on the human chondrocyte viability. A, Chemical construction of ATG. (B,C), The cytotoxic effect of ATG (0, 5, 10, 50 and 100 μmol/L) on human chondrocytes for 24 and 48 h was assessed using the CCK‐8 assay. Data represented are the means ± standard deviation (S.D). *P < .05, **P < .01, vs ATG (0 μmol/L), n = 5

The chemical structure of ATG is presented in Figure 1A. The cytotoxic effects of ATG (0, 5, 10, 50 and 100 μmol/L) on chondrocytes were determined by CCK‐8 assay. No cytotoxic effect of ATG was found at the dose of 5‐50 μmol/L (*P* < .01). Whereas the concentration increased to 100 μmol/L, the cell viability was decreased at both 24 hours (*P* = .0261) and 48 hours (*P* = .0062). Therefore, related lower concentration of ATG (5, 10 and 50 μmol/L) was utilized for the following experiments.

### 3.2 Arctigenin suppressed the IL‐1β and increased PGE2, IL‐6, NO, iNOS, TNF‐α and COX‐2 levels in chondrocytes

**Figure 2:**
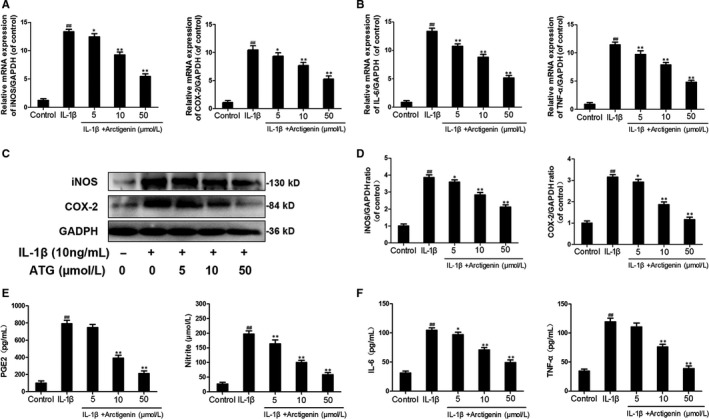
Arctigenin (ATG) decreased the IL‐1β–stimulated inflammatory cytokines. The human chondrocytes were pre‐treated with ATG (5, 10, 50 μmol/L) before the IL‐1β irritation. (A,B), The expressions of IL‐6, iNOS, TNF‐α and COX‐2 in mRNA level were assessed by the means of RT‐PCR. (C,D), The protein level and quantification analysis of iNOS and COX‐2 were measured by Western blot. (E,F), The protein level of IL‐6, TNF‐α, PGE2 and NO was assessed by ELISA. Data represented are the means ± SD #*P* < .05, ##*P* < .01, vs control group; *P < .05,**P < .01, vs IL‐1β–alone treatment group, n = 5

The efficacy of ATG on the expression of iNOS and COX‐2 induced by IL‐1β in human chondrocytes was detected through qRT‐PCR and Western blotting. IL‐1β greatly up‐regulated both the mRNA and protein levels of iNOS (13.35‐fold in mRNA and 3.86‐fold in protein) and COX‐2 (10.46‐fold in mRNA and 3.15‐fold in protein). However, although the ATG‐treated group still showed higher levels of iNOS and COX‐2 compared with untreated control (*P* < .01), it could intensively decrease this inflammatory reaction at least a half to the IL‐1β group (Figure 2A,C,D). At the same time making a contrast with the control group, the IL‐1β–induced group displayed up‐regulation of IL‐6 and TNF‐α at both mRNA and protein levels (IL‐6:13.32‐fold in mRNA and 3.34‐fold in protein; TNF‐α: 11.41‐fold in mRNA and 3.45‐fold in protein). Not surprisingly, however, the adverse effects were reversed by the pre‐treatment with ATG in a dose‐dependent manner, and the 50 μmol/L of ATG group decreases these levels to 1.5‐5.1‐fold of control (Figure 2B,F). Moreover, application of ATG dramatically exhibited an inhibitory effect on the endogenous NO and PGE2 increases in a dose‐dependent manner (decrease to around twofold of control), which were up‐regulated by around sevenfold of control after IL‐1β stimulation (Figure 2E). The results in this chapter indicate that ATG prominently inhibited inflammatory factor levels in human OA chondrocytes with IL‐1β stimulation.

### 3.3 Arctigenin enhanced ECM synthesis and reduced ECM degradation induced by IL‐1β in chondrocytes

**Figure 3:**
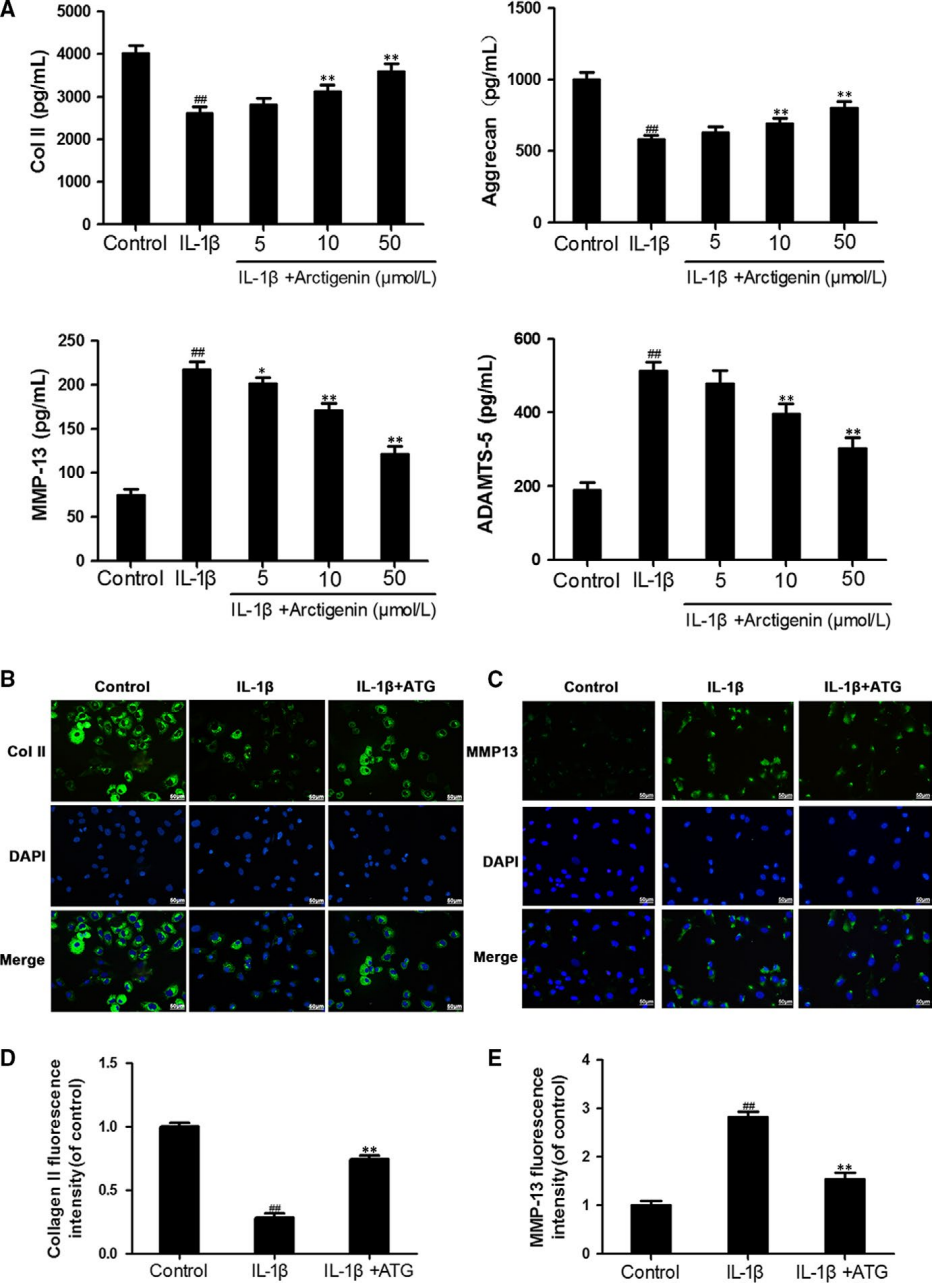
Arctigenin (ATG) shows a protective effect on the ECM metabolism. The human chondrocytes were pre‐treated with ATG (5, 10, 50 μmol/L) before the IL‐1β irritation. A, The protein level of Col II, MMP‐13 aggrecan and ADAMTS‐5 from human OA chondrocytes was detected by ELISA. (B,C), The representative fluorescence image of collagen II and MMP13 with DAPI (nuclei); scale bar: 50 μm. (D,E), The ImageJ software was used to quantify the fluorescence intensity of MMP‐13 and collagen II. Data represented are the means ± SD #*P* < .05, ##*P* < .01, vs control group; *P < .05,**P < .01, vs IL‐1β–alone treatment group, n = 5

Next, to determine the ATG on ECM synthesis and degradation under the stimulation of IL‐1β, we investigate the expression of CoI II and aggrecan, with or without ATG, and the production of MMP‐13 and ADAMTS‐5. The up‐regulation of MMP‐13 (2.9‐fold of control) and ADAMTS‐5 (2.7‐fold of control) attributed to IL‐1β activation was highly inverted by ATG pre‐treatment (about 1.5‐fold of control in 50 μmol/L of the ATG group). On the contrary, according to Figure 3A, type II collagen and aggrecan synthesis were down‐modulated by the stimulation of IL‐1β (decreased to 65% and 58% of control, respectively) but improved by ATG to 89% and 80% of control, respectively. All of these protective effects of ATG were in a dosage‐dependent manner (5, 10, 50 μmol/L). In addition, the results of immunofluorescence imaging of collagen II and MMP‐13 were in no conflict with the ELISA results (Figure 3B,C). Taken together, these results indicate that ATG alleviates ECM degradation and promotes ECM synthesis in IL‐1β–induced chondrocytes.

### 3.4 Arctigenin attenuated IL‐1β–induced NF‐κB activation in chondrocytes

**Figure 4:**
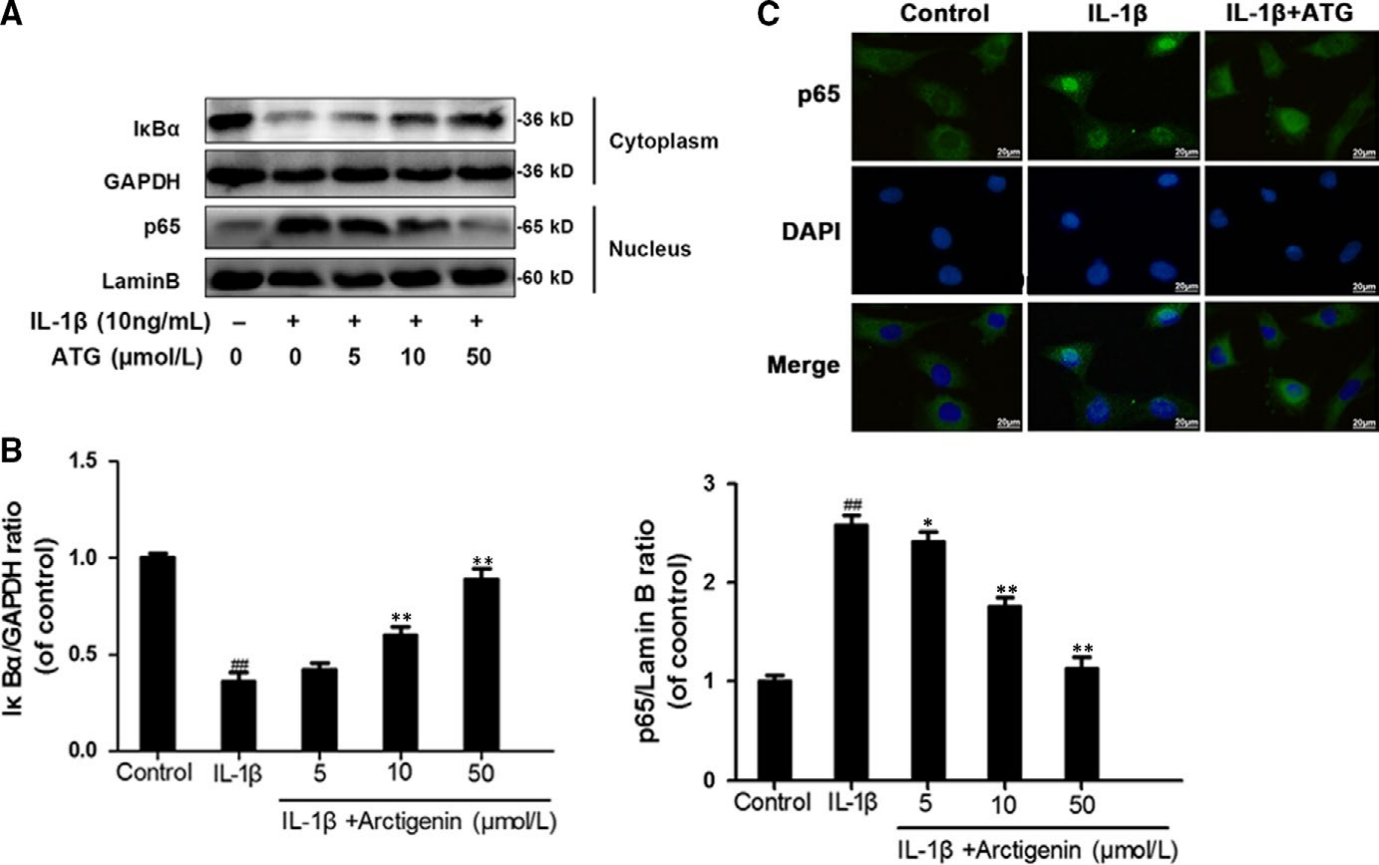
Arctigenin (ATG) attenuated IL‐1 β–activated NF‐κB signalling pathway. (A,B), The protein level of IκBα in cytoplasm and p65 in nucleus was detected by Western blotting. C, The representative fluorescence image of p65 with DAPI (nuclei); scale bar: 10 μm. Data represented are the means ± SD #*P* < .05, ##*P* < .01, vs control group; *P < .05,**P < .01, vs IL‐1β–alone treatment group, n = 5

To assess the role ATG played in the NF‐κB signalling pathway, we firstly separated the nuclear and cytosol proteins and then utilized Western blotting to examine the protein levels of IκBα in cytoplasm and p65 in nuclei, respectively. As is shown in Figure 4A,B, IL‐1β markedly promoted the degradation of IκBα (decrease to 36% of control) in the cytoplasm and more dissociated p65 was phosphorylated (up‐regulated to 2.58‐fold of control) and be freed to translocate from cytoplasm to nucleus. However, ATG pre‐treatment highly inhibited these changes. The IκBα level was recovered to 89% of control, and the p65 level in nucleus was down to 1.13‐fold of control in 50 μmol/L of the ATG group. Furthermore, we carried out experiments to measure the nuclear translocation of NF‐κB p65 via the immunofluorescence staining of p65 (Figure 4C). This experiment contained three groups: (a) control group, (b) IL‐1β group and (c) IL‐1β + ATG (50 μmol/L) group. Making a comparison between experimental groups and control group, the result shows that the p65‐active proteins were transferred from the cytoplasm to the nucleus followed by IL‐1β stimulation. However, ATG pre‐treatment sharply decreases this motion. In brief, the findings from this study provide a new understanding that the pre‐treatment of ATG highly inhibits IL‐1β–induced NF‐κB activation.

### 3.5 Arctigenin inhibited the PI3K/Akt signalling pathway induced by IL‐1β in human OA chondrocytes

**Figure 5:**
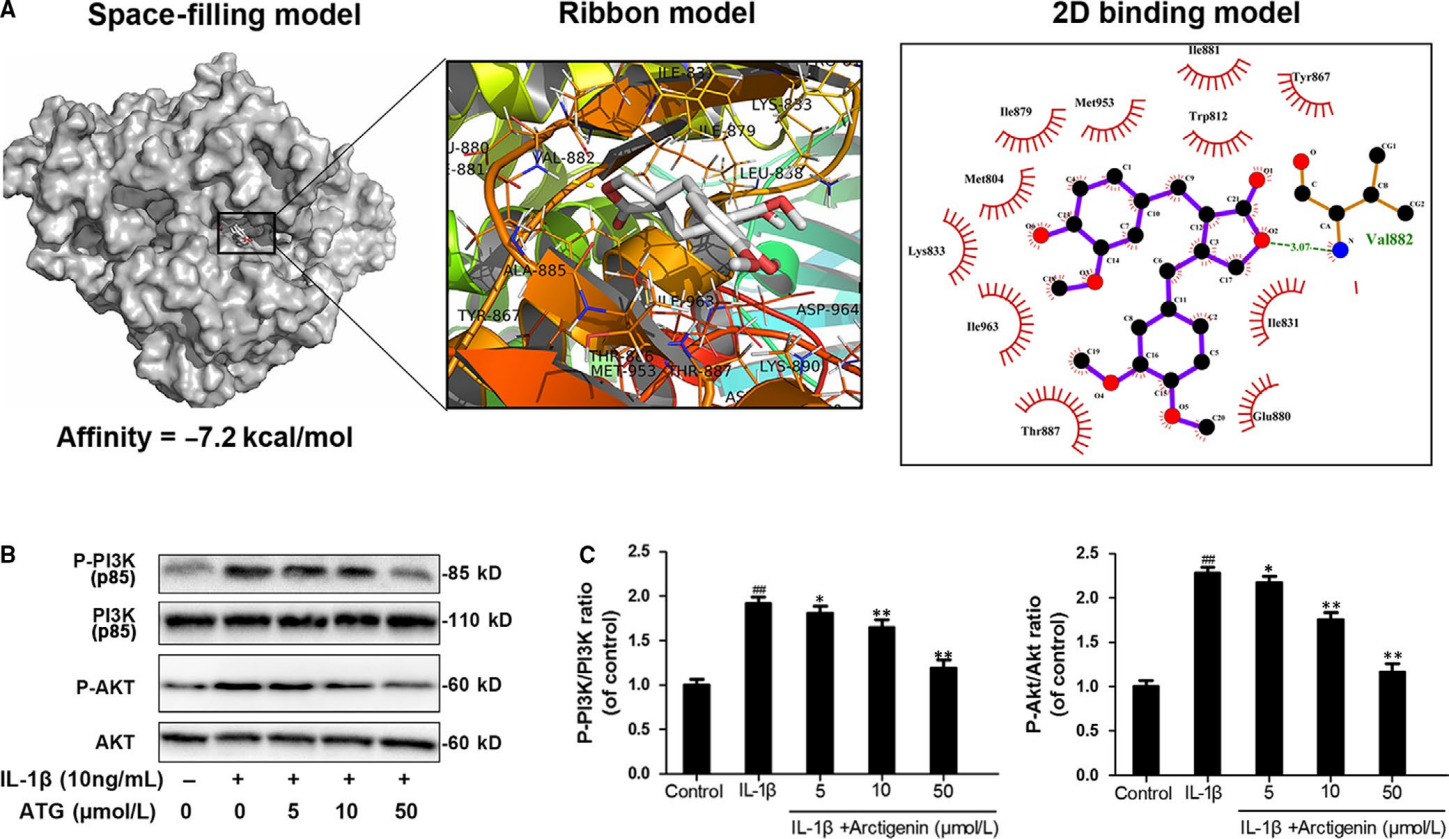
Arctigenin (ATG) inhibits the IL‐1β–activated PI3K/Akt signalling pathway. A, Molecular docking study of ATG binding to the inhibitory pocket of PI3K. (B,C), The phosphorylation level of PI3K and AKT was measured via Western blotting. Data represented are the means ± SD #*P* < .05,##*P* < .01, vs control group; *P < .05,**P < .01, vs IL‐1β–alone treatment group, n = 5

The association of upstream axis of NF‐κB with ATG was firstly examined by performing docking analysis of ATG with the antagonist binding site of PI3K.^55^ According to all returned molecular models, ATG was found to exert wonderful interaction with PI3K (Figure 5A). A space‐filling model illustrated that ATG was perfectly embedded in the inhibitory pocket of the crystal structure of PI3K. Similarly, the local interaction between ATG and related protein residues was revealed by the image of ribbon model and local 2D binding model. After simulated in AutoDock tools, a high affinity of −7.2 kcal/mol was calculated and a hydrogen bond formed between ATG and amino acid residues (VAL882) of PI3K was uncovered. Depending on the Ligplus, we can easily see the hydrophobic interaction was formed around ATG with the residue of Try867, Ile881, Tyr812, Met953, Ile879, Met804, Lys833, Ile963, Thr887 and Glu880 of PI3K. On the other hand, according to Western blotting, ATG pre‐treatment intensively inhibit the IL‐1β–stimulated PI3K and Akt phosphorylation in a dose‐dependent manner. The phosphorylation levels of PI3K and Akt were increased to 1.92‐ and 2.28‐fold of control by IL‐1β, but decreased to 1.13‐ and 1.16‐fold of control in 50 μmol/L of the ATG group (Figure 5B,C). To sum up, these results demonstrated that the pre‐treatment with ATG inhibits activation of PI3K/Akt axis.

### 3.6 Arctigenin ameliorated OA development in a mouse DMM model

**Figure 6:**
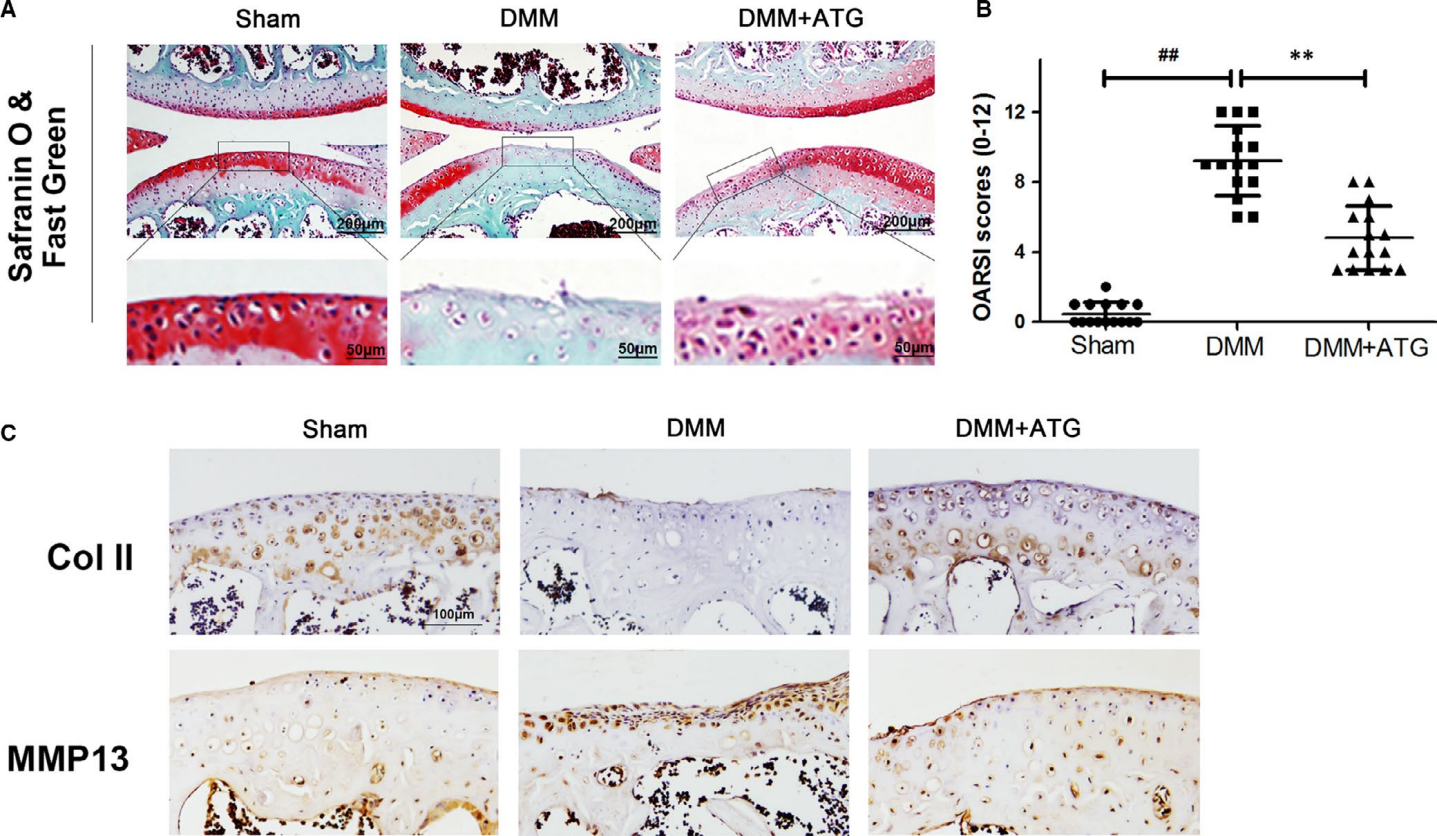
Arctigenin (ATG) inhibits OA development in mice. A, Typical Safranin O staining of the cartilage and subchondral cortical bone (scale bar: 200 μm or 50 μm). B, Diagrams show the standard cartilage OARSI scores. C, Immunohistochemistry staining of collagen II and MMP13 (scale bar: 100 μm). Data represented are the means ± SD #*P* < .05, ##*P* < .01, vs control group; *P < .05,**P < .01, vs IL‐1β–alone treatment group, n = 5

The therapeutic action of ATG in vivo was also confirmed in DMM model. As illustrated in the image of Safranin O/Fast Green staining, results indicated that the cartilage erosion, hypocellularity and a high proteoglycan loss were clearly presented in the DMM group compared with the sham one (Figure 6A). Nevertheless, oral administration of ATG (30 mg/kg) could mitigate these detrimental effects. The OARSI scores were also applied for quantitative analysis (Figure 6B). Altogether, these results indicate that ATG ameliorates OA development in vivo*.*

## 4 DISCUSSION

At present, according to the OARSI guidelines of non‐surgical management of OA published in 2019, most therapies are mainly emphasizing on the early prevention and symptom relief, such as habit adjustments (eg agility training, weight loss in obese patients and regular exercises) and symptomatic treatment by drugs (eg paracetamol and nonsteroidal anti‐inflammatory drugs).^28^ All of the drug interventions are limited to pain relief. While in the late stage of the disease, no treatments are effective enough to slack down the development of the disease besides surgical procedure (total joint replacement).^56^, ^57^ NSAIDs, especially, though prevalently used for the treatment of OA, are not only deficient in lacking steady effectiveness but also give rise to quite a few severe side effects during constant appliance.^58^ Similar to NSAIDs, chondroitin sulphate and glucosamine were demonstrated to help to pain‐relief. However, their effects to protect against OA development were still not verified.^29^, ^32^ Hence, although OA as a degenerative joint disease cannot be reversed completely, researches targeting on a safer and milder drug development make a vital contribution to the prevention of OA. For the aim of solving such urgent problems, compounds based on natural products have become the focal points for the OA prevention by possessing the strong anti‐inflammatory efficacy and minimal side effects.^59^

Arctigenin, one component of dietary vegetables (greater burdock) in Asia, especially China, Korea and Japan, has therapeutic effect on anti‐influenza, antioxidant, antiviral, anti‐inflammatory, neuronal protection and anticancer treatment. 34, 35, 36, 37, 38 However, no previous studies have clarified its effects and underlying mechanisms on the treatment of OA. Thus, the current study sheds new light on the potential protective role in OA both in vitro and in vivo.

The therapeutic agent aiming to disturb the secretion of MMP13 and ADAMTS5 seems viable for the treatment of OA.^29^ Based on our current research, we demonstrated that NF‐κB activation up‐regulated above‐mentioned inflammatory mediators can be attenuated via the pre‐treatment of ATG in chondrocytes, which was consistent with the research from Liu, H et al, who found that ATG owns a protective effect on human rheumatoid arthritis fibroblast‐like synoviocytes via suppressing the Akt/NF‐κB axis.^44^ Thus, our results give another potent evidence to support ATG in arthritis‐related disease application.

However, in the aspect of mechanism of ATG, we do not believe that the ATG owns its protective functions through targeting NF‐κB directly. At this account, it is of full value for its upstream signalling to be detected. Previous study demonstrated that PI3K is one of the most upstream intracellular signalling transductors in PI3K/AKT axis, which directly responds to the activation of membrane receptor of stimuli, such as cytokines, growth factors, pro‐inflammatory mediates.^25^, ^26^, ^27^ Inhibition of PI3K/Akt signalling pathway may reduce cartilage degeneration via multiple downstream targets, among which NF‐κB is considered as the most significant one.^26^ Hence, PI3K/Akt/NF‐κB axis can be considered as a potential therapeutic target for treating OA.^26^ As the docking analysis shows, we found that ATG strongly binds to the inhibitory pocket of PI3K. Accordingly, the IL‐1β–induced PI3K/Akt phosphorylation was inhibited by ATG intervention, which was in agreement with the previous study, showing that pre‐treatment with ATG significantly suppressed PI3K/Akt phosphorylation and NF‐κB activation in LPS‐stimulated peritoneal macrophages and on LPS‐induced systemic inflammation and 2,4,6‐trinitrobenzene sulphonic acid (TNBS)–induced colitis in mice.^39^ Thus, the inhibitory effects of ATG on NF‐kB activation were followed by inhibition of PI3K/AKT. Although many other signalling molecules might also participate in ATG‐induced protective effects in OA, we did not exclude or verify all potential factors one by one in the present study. However, it is our directions in future's works.

To further investigate the protective effect of ATG in vivo, we launched an animal model of OA in the present work. DMM is a highly authentic and efficient method to make an animal OA model for the in vivo analysis. Equally for OA patients, the DMM mice featured with a damaged cartilage surface, hypocellularity and a high proteoglycan loss. However, all the histological changes were attenuated by the pre‐treatment of ATG. In addition, compared with the DMM group, the ATG pre‐treatment group had lower OARSI scores. These results above were in accordance with the in vitro work and suggested that ATG was safe and effective in vivo*.*

**Figure 7:**
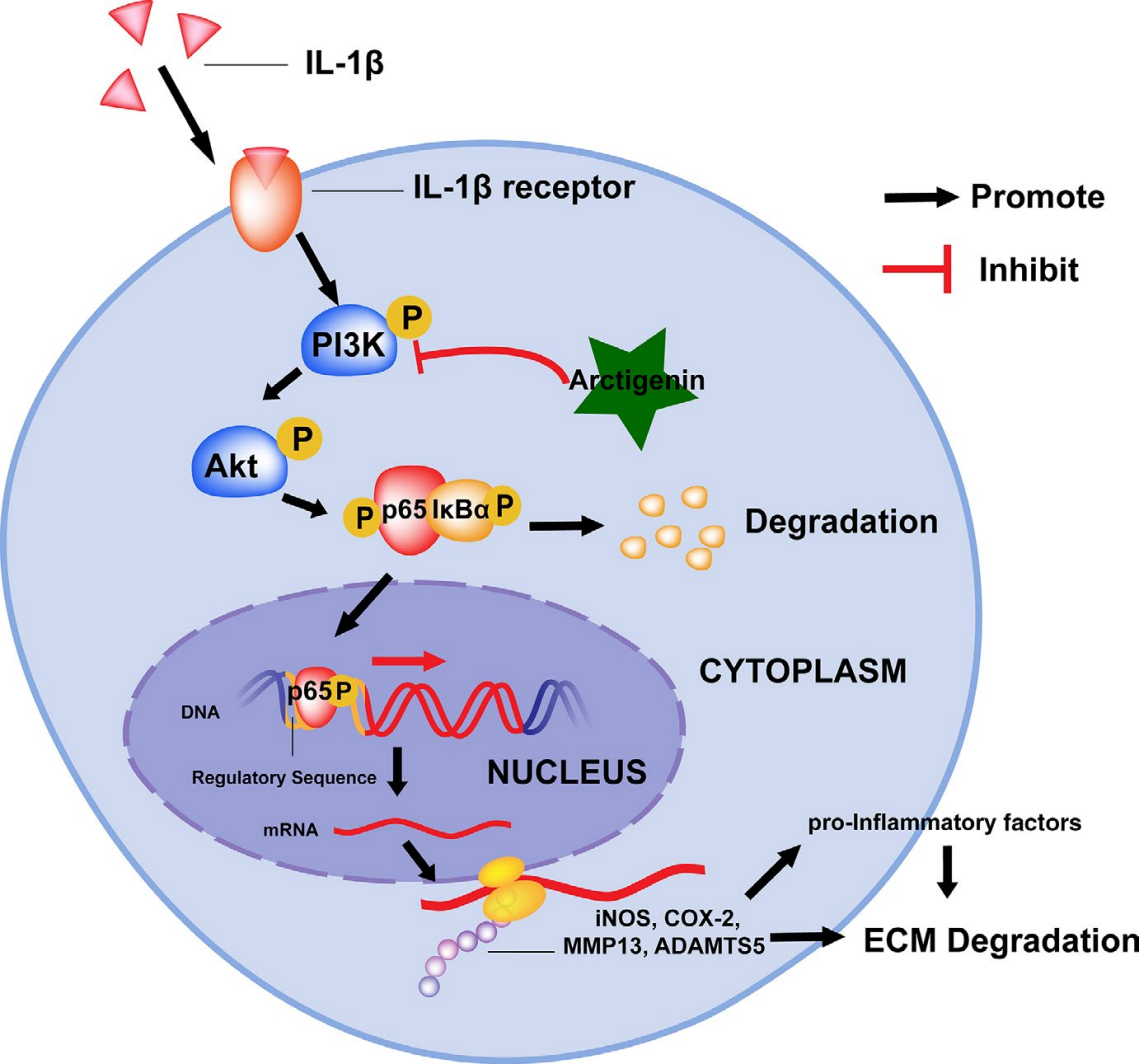
Schematic illustration of PI3K/Akt/NF‐κB suppression by ATG and its potential protective effects against IL‐1β in OA development

In conclusion, this is the first study that demonstrates ATG is a potent natural agent for OA protection via inhibiting the PI3K/Akt/NF‐κB axis and the latent molecule mechanism is displayed specifically in Figure 7. Although no significant improvements were noted in comparison with ATG‐treated group with the healthy control at present data, ATG treatment could attenuate the progression of OA in pathological condition both in vitro and in vivo, indicating its potential as a prophylactic treatment option of OA.

## CONFLICTS OF INTERESTS

The authors confirm that there are no conflicts of interest.

## AUTHOR CONTRIBUTIONS

HXL, WJZ and SKT designed the research study. JKZ, SYY and SKT found and read relevant literatures. HXL, PS and SKT designed the experiments. WJZ, SKT, XYZ, JCX, HSH, JKZ and XNZ performed experiments. WJZ, XYZ, JCX and XNZ analysed data to form graphs. SKT and WJZ wrote the manuscript. HXL, QT and PS helped to write and modified the manuscript. QT helped to the revision of the manuscript.

## Data Availability

The data used to support the findings of the study are available from the corresponding author upon request.
